# Primary Somatosensory Cortices Contain Altered Patterns of Regional Cerebral Blood Flow in the Interictal Phase of Migraine

**DOI:** 10.1371/journal.pone.0137971

**Published:** 2015-09-15

**Authors:** Duncan J. Hodkinson, Rosanna Veggeberg, Sophie L. Wilcox, Steven Scrivani, Rami Burstein, Lino Becerra, David Borsook

**Affiliations:** 1 Center for Pain and the Brain, Department of Anesthesiology, Perioperative & Pain Medicine, Boston Children’s Hospital, Harvard Medical School, Boston, MA, United States of America; 2 Department of Oral and Maxillofacial Surgery, Massachusetts General Hospital, Boston, MA, United States of America; 3 Anesthesia and Critical Care, Beth Israel Deaconess Medical Center, Harvard Medical School, Boston, MA, United States of America; 4 Department of Psychiatry, McLean Hospital, Harvard Medical School, Boston, MA, United States of America; Taipei Veterans General Hospital, TAIWAN

## Abstract

The regulation of cerebral blood flow (CBF) is a complex integrated process that is critical for supporting healthy brain function. Studies have demonstrated a high incidence of alterations in CBF in patients suffering from migraine with and without aura during different phases of attacks. However, the CBF data collected interictally has failed to show any distinguishing features or clues as to the underlying pathophysiology of the disease. In this study we used the magnetic resonance imaging (MRI) technique—arterial spin labeling (ASL)—to non-invasively and quantitatively measure regional CBF (rCBF) in a case-controlled study of interictal migraine. We examined both the regional and global CBF differences between the groups, and found a significant increase in rCBF in the primary somatosensory cortex (S1) of migraine patients. The CBF values in S1 were positively correlated with the headache attack frequency, but were unrelated to the duration of illness or age of the patients. Additionally, 82% of patients reported skin hypersensitivity (cutaneous allodynia) during migraine, suggesting atypical processing of somatosensory stimuli. Our results demonstrate the presence of a disease-specific functional deficit in a known region of the trigemino-cortical pathway, which may be driven by adaptive or maladaptive functional plasticity. These findings may in part explain the altered sensory experiences reported between migraine attacks.

## Introduction

The human brain is exquisitely sensitive to changes in cerebral blood flow (CBF). Under normal resting conditions, it has been shown that regional CBF (rCBF) is closely coupled with glucose utilization, oxygen consumption, and aerobic glycolysis [1, 2]. This relationship is thought to be regulated locally and dynamically by neurons and astrocytes [3], with direct autonomic control of the cerebral vasculature [4]. At the systems level, there is a potential coupling between CBF and brain functional network topology [5–7]. Therefore, the human brain exhibits a hierarchical organization during rest, the maintenance of which demands the majority of the brain’s metabolic energy and blood flow [8].

Migraine is a complex disorder that is accompanied by significant derangements in neurovascular function [9–11]. This dysfunction may have important consequences for resting brain perfusion, as minor disruptions in CBF could reflect subtle changes in metabolism resulting from the underlying condition. Perturbations of brain metabolism have been demonstrated both before and during migraine attacks [12, 13], indicating a possible increase in energy demands caused by neuronal hyperexcitability [14–16]. In addition, direct immuno-vascular interactions and autonomic dysfunction may act as potential modulators of the cerebral circulation [4, 17]. The presence of altered metabolism or energetic dysfunction, together with regional perfusion deficits, could indicate the vulnerability of specific neuronal populations.

Human imaging studies have shown varying effects on CBF in the different phases of migraine headache (reviewed in [18]). A small proportion of these findings represent CBF changes that are not related to the migraine attack itself, as the data were collected interictally [19–23]. Notwithstanding the contributions of these studies, several methodological factors limit their scope and applicability. For example, the implementation of perfusion imaging has exclusively relied upon methods that involve the inhalation or injection of radioactive tracers or contrast agents, which limits its repeatability and application in healthy volunteers. Additional sources of variation may be attributed to the clinical inclusion criteria, symptomatology, and attack characteristics of migraine patients [24]; hence the effects reported in these studies might not be directly comparable.

In this study we used the magnetic resonance imaging (MRI) technique—arterial spin labeling (ASL)—to non-invasively and quantitatively measure rCBF in a case-controlled study of migraine. ASL provides superior spatial resolution and sensitivity relative to traditional nuclear functional imaging techniques [25, 26]; as such, we hypothesized that the alterations in rCBF might in fact be more localized than has been previously reported in migraine, specifically affecting those brain regions known to be implicated in processing trigeminal nociceptive information [27]. It is noteworthy that those cortical and subcortical regions may have a high capacity for neuroplasticity, therefore basal differences in brain perfusion may help distinguish brain regions that are more vulnerable to repetitive migraine attacks.

## Materials and Methods

### Ethical approval and consent

The Institutional Review Board at McLean Hospital, Harvard Medical School, approved the study. All experiments fulfilled the criteria of the Helsinki accord for human research. Informed written consent was obtained from all participants.

### Participants

Thirty-four right-handed participants were enrolled in this study: 17 migraine patients (mean age ± SD; 28±9.1, range 19–48 years, 5 male/ 12 female) and 17 healthy age- and sex-matched controls. To minimize variability in the clinical inclusion criteria, symptomology, and attack characteristics [24]; we restricted the patient selection to migraine without aura. The migraine patients had to meet the following criteria: (i) episodic migraine as classified in the International Classification for Headache Disorders, second edition (ICHD-II); (ii) had suffered from episodic migraine for ≥3 years; and (iii) have no migraine 72 h prior to the study session and no symptoms of developing a migraine during or 24h after the scans. Patients were excluded if they had chronic migraine or were taking daily medication including prophylactic migraine treatment. Healthy controls were excluded if they had any type of migraine or first-degree relatives with a history of any type of migraine. Females were excluded if they were pregnant.

### Demographic and headache characteristics

Retrospective migraine attack characteristics were collected on inclusion (see Table 1). Patients were also asked to complete the Allodynia Symptom Checklist, assessing the frequency of allodynia symptoms during headache. The questionnaire was as follows: Do you experience pain or unpleasant sensation *on your skin* during a migraine attack when you engage in any of the following activities (Yes, No, or Not applicable): (i) combing your hair; (ii) pulling your hair back (e.g. ponytail); (iii) shaving your face; (iv) wearing eyeglasses; (v) wearing necklaces; (vi) taking a shower; (vii) resting your head on a pillow; (viii) being exposed to heat (e.g. standing next to a stove while cooking); (ix) being exposed to cold (e.g. breathing through your nose on a cold day); (x) wearing tight clothes; (xi) wearing a watch or bracelets [28–30].

**Table 1:** Clinical details and migraine history. Abbreviations: Age, age in years; Sex, male (M)/female(F); Age at onset, age the headaches started in years; Frequency, number of attacks per month (median values are given in brackets); Duration, duration of illness in years; Side, dominant side of headache pain, left(L)/right(R); Allodynia, patients reporting at least one type of skin hypersensivity during migraine.

Subject	Age	Sex	Age at onset	Duration	Frequency	Side	Allodynia
M01	25	F	15	10	12	L & R	Yes
M02	48	F	25	23	1–3 (2)	L	No
M03	24	F	20	4	1	L	Yes
M04	26	M	23	3	9	R	Yes
M05	30	F	21	9	2	R	Yes
M06	38	F	11	27	4–8 (6)	L	Yes
M07	38	F	26	12	1–2 (1.5)	L	Yes
M08	19	F	12	7	4–10 (7)	L & R	Yes
M09	19	F	14	5	0.3	L	Yes
M10	18	M	8	10	2	L & R	Yes
M11	21	M	6	15	3–4 (3.5)	R	No
M12	45	F	15	30	4	R	Yes
M13	29	M	17	12	3–4 (3.5)	L	No
M14	23	F	13	10	9	L	Yes
M15	23	F	13	10	2–3 (2.5)	L & R	Yes
M16	33	M	24	9	4	L & R	Yes
M17	23	F	13	10	4–6 (5)	R	Yes

### MRI acquisition

Participants were scanned in supine position on a Siemens Magnetom 3T whole-body MRI scanner (Siemens Healthcare Inc., USA) using a 12-channel head coil. For image registration purposes, high-resolution T1-weighted anatomical scan was acquired using a 3D MPRAGE sequence [FOV = 256 mm^2^, TR/TE = 2000/3.5ms, 256×256 matrix, 224 slices, voxel size 1x1x1mm]. Perfusion measurements were performed using pseudo-continuous arterial spin labeling (pCASL) [31, 32]. Arterial blood was labeled using a 1.5sec train of RF pulses and a single post-labeling delay (PLD) time of 1.3sec. The offset distance of the labeling plane was positioned 90mm beneath the center of the acquired slices (parallel to the AC-PC line), ensuring optimal labeling of the posterior cerebral circulation [33]. Imaging readout was multi-slice single-shot GE-EPI [TR/TE = 3870/12, FOV = 220mm^2^, matrix = 64x64, 26 slices acquired in ascending order (no slice gap), slice thickness = 5mm, slice acquisition time = 41.9ms]. For signal averaging purposes, 30 tag-control pairs of images were collected in succession, corresponding to 4.01 minutes. Two reference calibration images (no labeling or background suppression, TR = 7sec, all other parameters identical to pCASL scan) were collected to enable the estimation of the equilibrium magnetization of arterial blood. All participants were imaged under identical conditions with eyes closed.

### Quantification of cerebral blood flow

The pCASL data were motion corrected for the control and label image series separately. To minimize blood-oxygen-level-dependent (BOLD)-contamination, the tag-control difference images were calculated using surround subtraction [34], and fitted to the single-compartment Standard Kinetic Model [35]:

$$CBF\;=\;\frac{6000\;\cdot \;\lambda \;\cdot \;R_{1a}}{2\;∙\;\alpha \;∙\;({(e}^{-\omega R_{1a}})-\;{(e}^{-(\tau +\omega )R_{1a}}))}\;∙\;\frac{\Delta M\;}{\;M_{0}\;}\;=\;Z\;∙\;\frac{\Delta M\;}{\;M_{0}\;}$$

where *λ* is the blood-brain partition coefficient, *ΔM* is the difference signal between the control and label acquisitions, *R1a* is the longitudinal relaxation rate of blood, *τ* is the labeling time, *ω* is the post labeling delay time, *α* is the labeling efficiency, and *M0* is the mean signal intensity of the two calibration images. The parameters used in this study were *R1a* = 0.67 sec^-1^[36], *α* = 0.85 [31], *λ* = 0.9 mL/g [37], *τ* = 1.5 sec, *ω* = 1300 msec. The parameter *Z* accounts for the serial acquisition of slices. The transformation of CBF to absolute units (ml/100g/min) was performed in native space.

Implementation of the pCASL sequence and absolute CBF quantification follows current recommendations for the optimal use of ASL in clinical populations [38]. Further details of the retest reliability of the pCASL technique have been established previously [39].

### Image post-processing

Imaging data were processed using SPM8 (http://www.fil.ion.ucl.ac.uk/spm). The high-resolution structural images were used to align the CBF data to MNI152 standard space [average T1 brain image constructed from 152 normal subjects at the Montreal Neurological Institute (MNI), Montreal, QC, Canada]. Spatial normalization was performed by co-registering the mean motion-corrected ASL image to the T1 image. The spatial normalization parameters required to warp the T1 image to MNI space were estimated (via SPM unified segmentation) and these transformation parameters were applied to the CBF map. The data were spatially smoothed using a 6mm FWHM (full- width at half-maximum) Gaussian kernel to accommodate for gyral variability across subjects. Group-level voxel-wise changes in regional CBF were calculated under the framework of the general linear model (GLM) using a random effects independent two-sample t-test. Significant clusters were displayed with a probability threshold of *p*<0.05, corrected for multiple comparisons using family-wise error rate (FWE).

### Correlation between CBF and clinical variables

The linear dependence between the magnitude of the CBF and the patient’s clinical characteristics was examined using Pearson’s correlation coefficient.

## Results

### Demographic and headache characteristics

Patients reported a mean migraine (disease) duration of 12 years (±SD: 7.6, range: 3–30 years). The attack frequency ranged from 0.3–12 episodes per month (mean±SD: 4.8±4.1). Most patients reported unilateral pain (n = 5 right-sided, n = 7 left-sided), but some reported bilateral headache pain (n = 5). Patient characteristics are provided in Table 1. Importantly, there was a significant correlation between patients' duration of migraine and their age (r = 0.76, p = 0.0004).

**Fig 1:**
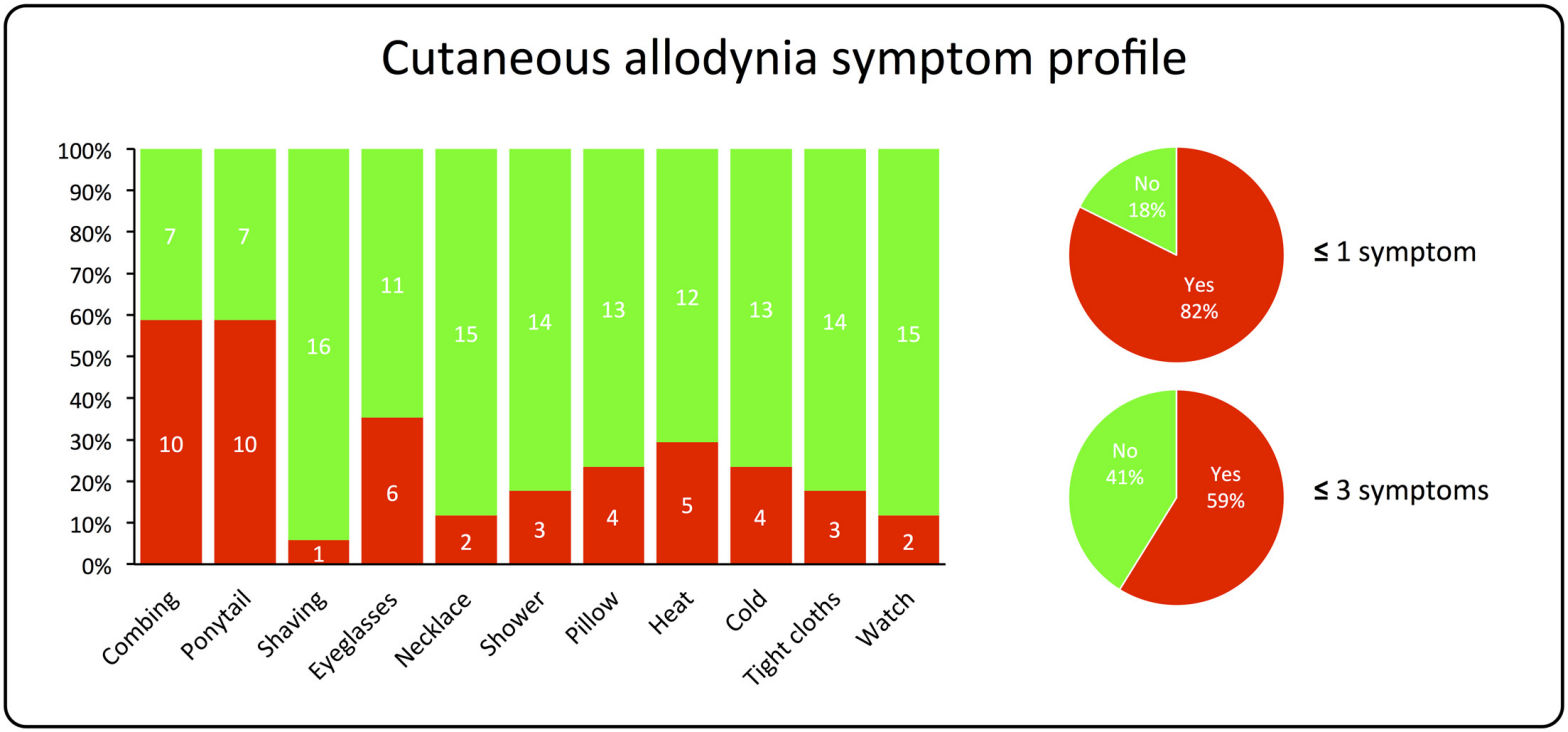
Cutaneous allodynia symptom profile. Proportion of total responses to individual questionnaire items of skin hypersensitivity **(bar chart)**. Inset small pie charts show the percentage of patients that reported ≤1 and ≤3 symptoms (yes = red), and those that were unaware of any abnormal skin sensitivity (no = green) [28–30].

Subjects were also asked to fill out a questionnaire to determine if they usually developed cutaneous allodynia during the migraine attack (Fig 1). A total 82% (14) of the patients reported to at least one type of skin hypersensitity during migraine, 18% (3) were unaware of any abnormal skin sensitivity. These proportions changed to 57% (10) reporting a minimum of three symptoms. The total number of definitive responses (yes) to individual questions varied greatly. Certain items in the questionnaire were sex specific (e.g. shaving, earrings), some items were not applicable to every patient (e.g. eyeglasses), and not every patient was able to reflect on whether a certain activity was bothersome during migraine (unsure).

### Group differences in rCBF

**Fig 2:**
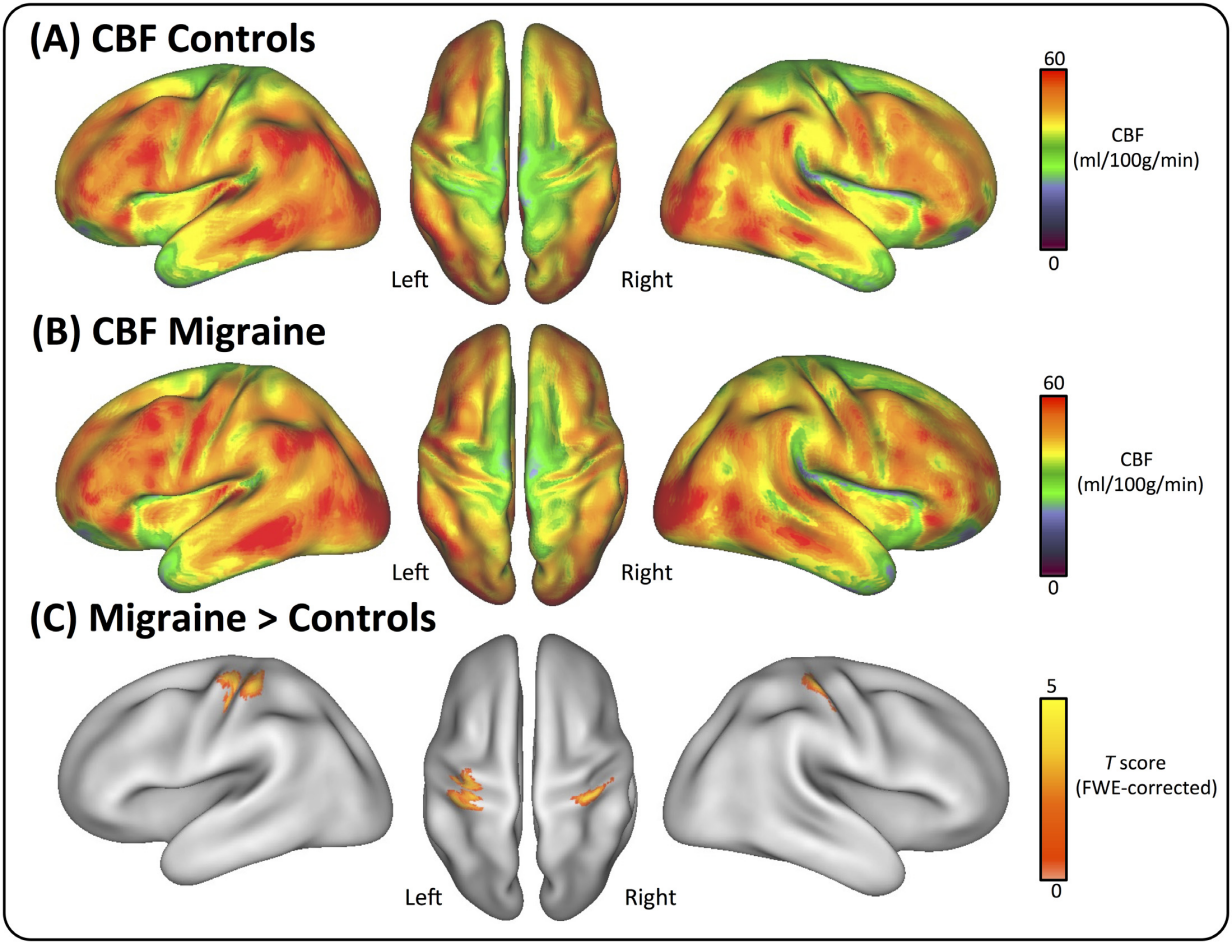
Distribution of regional cerebral blood flow (rCBF). The maps illustrate the quantitative CBF values (ml/100g/min) across all subjects in the control **(A)** and migraine groups **(B)**. Group-wise changes in rCBF are displayed in the bottom row **(C)**. Note the bilateral clusters of significantly increased rCBF in the primary somatosensory cortices (S1). Statistical images are displayed with a cluster probability threshold of P<0.05, corrected for multiple comparisons (FWE). Data are shown in Caret PALS space, with left/right orientations marked.

**Fig 3:**
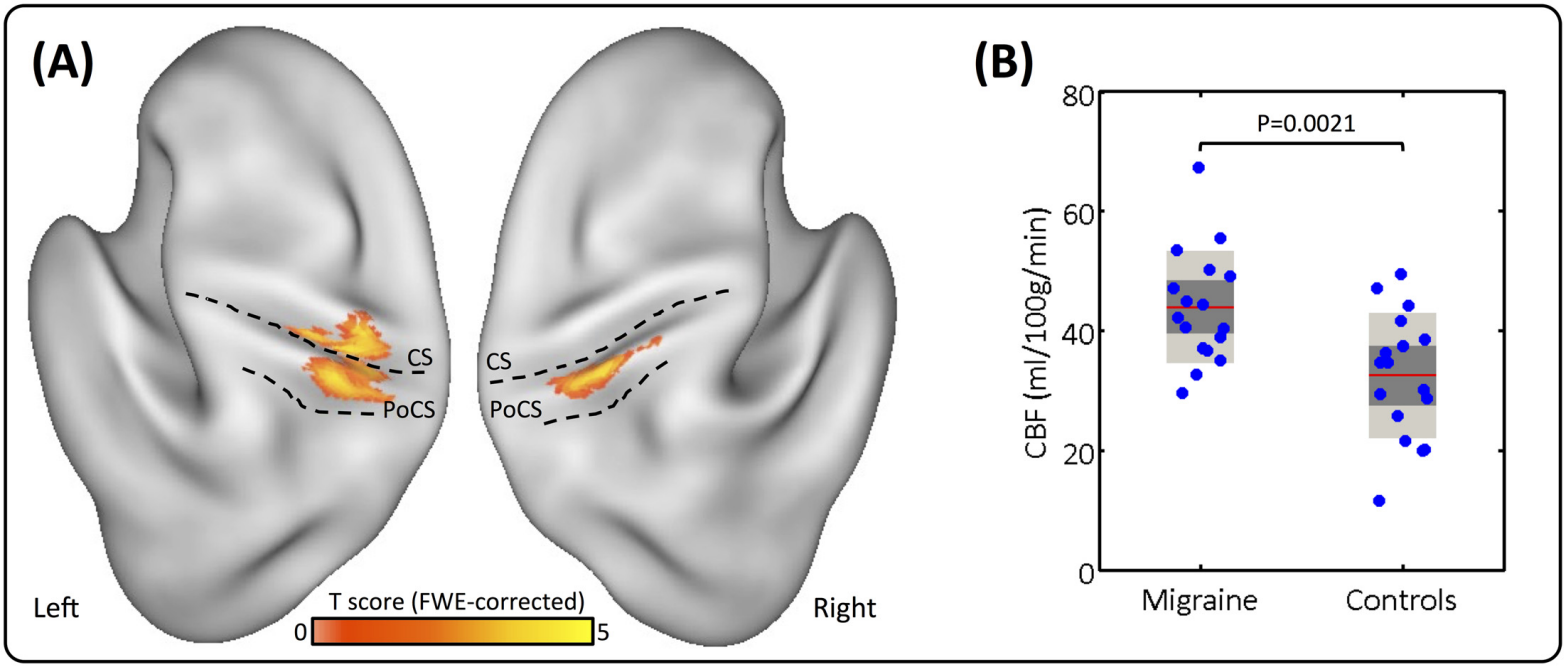
**(A)** Migraine-related increase of rCBF in the primary somatosensory cortices. Dotted black lines correspond to the boundary of central sulcus (CS) and post-central sulcus (PoCS). Statistical images are displayed with a cluster probability threshold of P<0.05, corrected for multiple comparisons (FWE). Data are shown in Caret PALS space, with left/right orientations marked. **(B)** Magnitude of the CBF changes within S1. Plots represent the mean (red line), 95% confidence interval (light-grey region), and 1 standard deviation (dark-grey region). Individual subjects data are shown in blue. Both groups are normally distributed, and significant after independent two-sample T-test (p = 0.0021).

Individual CBF maps were averaged over subjects in standard atlas space. The resulting distribution of regional CBF (rCBF) in the migraine patients and healthy controls is shown in Fig 2. Whole-brain statistical comparison revealed migraineurs had significantly higher rCBF in the somatosensory cortex than the controls. These CBF changes were localized to both the right and left post-central gyrus (extending into parts of the right inferior parietal cortex, right superior parietal cortex, and the left pre-central gyrus) (Fig 3). There were no significant regions of decreased perfusion (i.e. Migraine<Controls). Furthermore, there were no significant differences in the global CBF between patients and controls (independent two-sample t-test: *p* = 0.9338).

### Correlation between rCBF and headache characteristics

**Fig 4:**
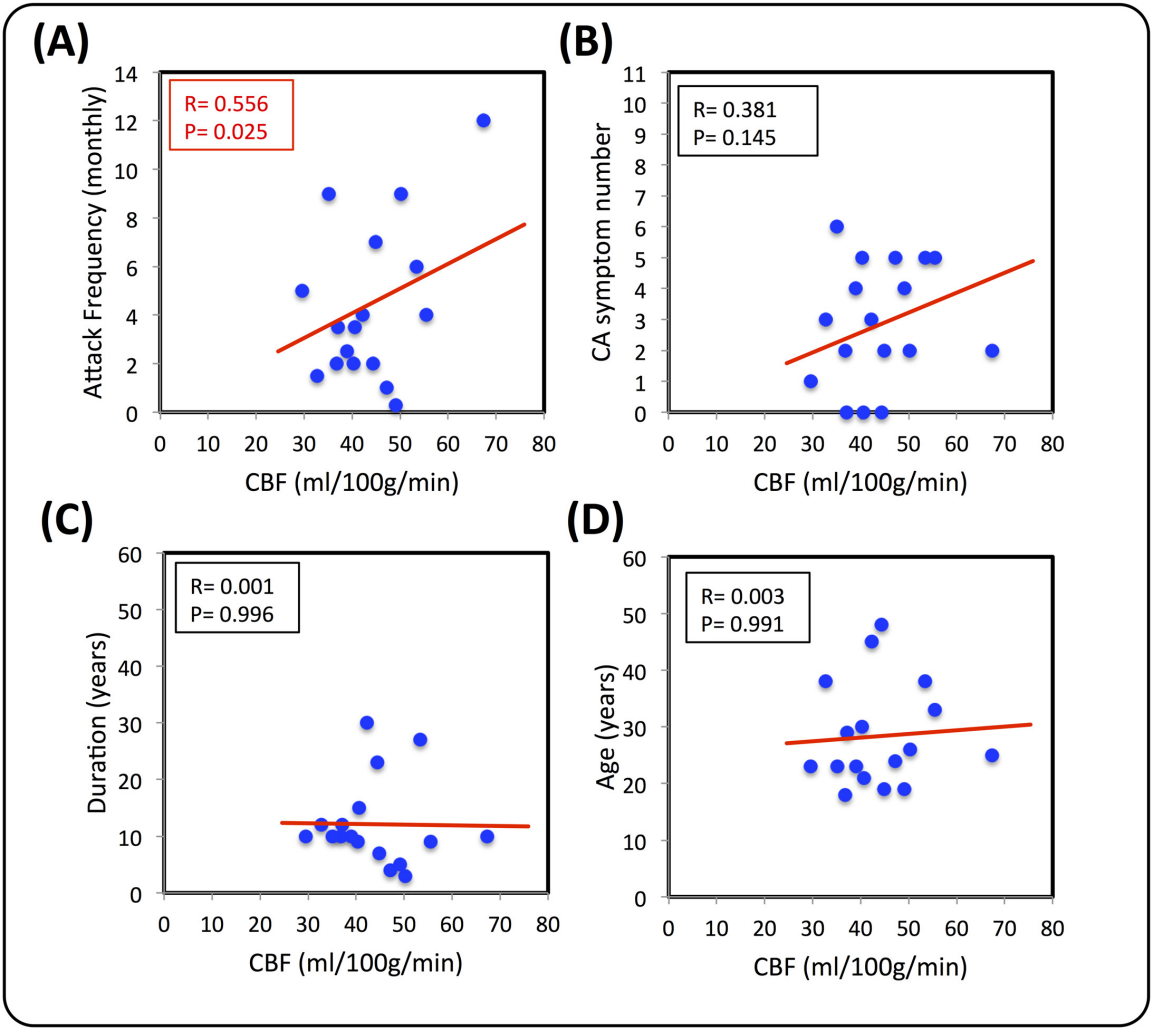
Correlations between rCBF and clinical reported variables. **(A)** Headache frequency (which was positively correlated with CBF in S1). **(B)** Total number of cutaneous allodynia (CA) symptoms during migraine, **(C)** Duration of illness, and **(D)** Age of the patients.

The results revealed a significant positive correlation between rCBF and the attack frequency (r = 0.556, p = 0.025) (Fig 4A), indicating patients with a higher number of attacks had higher levels of CBF within the S1 area. There were no significant correlations between CBF and the patients' total number of cutaneous allodynia (CA) symptoms (r = 0.381, p = 0.145), duration of migraine (r = 0.001, p = 0.996), or their age (r = 0.003, p = 0.991) (Fig 4B, 4C and 4D). We exclude the possibility of age-related effects on rCBF.

## Discussion

The present study demonstrates the utility of arterial spin-labeling (ASL) to derive non-invasive, quantitative measures of regional CBF in the migraine brain. Parenchymal perfusion is an important physiologic parameter in the evaluation and management of brain health as well as a surrogate index of neural activity. We observed regional variations in CBF in S1, and these changes were specifically related to the migraine attack frequency. The S1 area is strongly implicated in the ascending trigemino-cortical nociceptive pathway. We discuss the known functional plasticity of S1 and its possible interactions with altered sensory experiences in migraine.

### S1 changes in migraine

An exciting new finding of this study is the fact that migraine patients show increased rCBF in S1 compared to controls. Activation of the S1 region has been reported in approximately 75% of human imaging studies of pain [40]. It is also the earliest site to be activated in studies of laser-evoked pain [41], thus supporting a role for the S1 cortex in the sensory aspects of pain, including localization and discrimination of pain intensity. While the focus of this study is not on the migraine attack itself, the trigemino-cortical system remains an important pathway in migraine pathophysiology [27]. Migraine patients can present with a number of interictal functional abnormalities that are potentially related to the dysfunction of processing sensory information, including altered sensitivity to light, sound, smell, touch, and pain [42]. Patients often display a lack of habituation to repetitive sensory stimuli [15], which may relate to abnormal thalamo-cortical activity [43]. In response to the repeated attacks, there is also evidence of central sensitization in the trigeminal sensory pathway [44], which causes the clinical phenomenon of expanding cutaneous allodynia (CA) and hyperalgesia [45]. Somatosensory hypersensitivity and the development of CA occurs in around two-thirds of migraineurs during an attack [28, 29, 46], and approximately the same distribution of CA was reflected in our cohort of patients (see Fig 1). Interestingly, there was a positive (but not significant) linear trend between CBF in S1 and the number of reported CA symptoms during migraine (see Fig 4B). Collectively, these clinical and imaging findings support an underlying dysfunction in S1 and the trigemino-cortical pathway, which may cause vulnerability to migraine.

### Adaptive or maladaptive changes in S1

A second important finding of this study is the relationship between headache frequency and rCBF in the S1 area. Chronic or repetitive activation in response to heachache attacks may cause S1 to undergo plastic changes that can be induced by either peripheral or central inputs. Interestingly, the rCBF changes we observed were in the mid part of post-central gyrus, a location compatible with the hand/face area of the somatosensory homunculus [47]. While it may be the case that the receptive fields for this part of the body surface are spatially indistinguishable at the macroscopic level of the ASL signal; the CBF values did correlate positively with the attack frequency, thus suggesting headache pain may be driving adaptive or maladaptive functional plasticity that could be associated with increased local reorganization [48]. This hypothesis is not only supported by functional data, but also by structural changes that have been observed in migraine patients. For example, our group and others have demonstrated the presence of both grey and white matter changes in S1, which appear to track the ascending trigeminal nociceptive pathway [49–52]. Although the measurements derived from structural MRI are typically ambiguous in biological terms, it is possible that these functionally-related structural changes might explain the change in demand for CBF. However, direct evidence for synaptic remodeling will need to be confirmed from more invasive experiments.

### Variability of resting brain perfusion

To confidently compare CBF values across different cohorts of a population (i.e. migraine patients vs. healthy controls), it is important to consider the between subject variability. Several groups have conducted reliability studies of CBF measures employing ASL techniques [39]. The consensus is that ASL is comparable to other perfusion imaging techniques, such as positron emission tomography (PET) or single-photon emission computed tomography (SPECT). The mean grey matter CBF values for both migraine patients and healthy controls fall within the expected range for adults. However, the regional CBF estimates may be significantly affected by partial volume (PV) effects since any one voxel is unlikely to contain only a single tissue type [53–56]. The effects of PV are particularly problematic in areas that exhibit structural changes, such as tissue atrophy or morphology, thus we cannot exclude this potential complication in the migraine population [57]. It should also be considered that all patients had previously used acute and/or preventative medications which could theoretically alter CBF. However, as the global CBF values were not significantly different between the patients or controls, it is unlikely to be causing the effects [58]. Finally, the fact that we recruited migraine patients without aura and excluded patients with chronic headache could mean the results are not directly applicable to these groups, although there is no good evidence that these subtypes are fundamentally different in terms of pathophysiology.

### Conclusion

In this study we have shown that ASL is a versatile tool for investigating global and regional brain perfusion in migraine. We observed changes in CBF at an unprecedented level of spatial detail, and uncovered abnormal functioning in the primary somatosensory cortex (S1). The fact that the CBF values in S1 were positively correlated with attack frequency, but not the duration of illness or the age of the patients, suggests these effects may be driven by adaptive or maladaptive functional plasticity. Also, many of the patients reported skin hypersensitivity (cutaneous allodynia) during migraine, suggesting atypical processing of somatosensory stimuli. S1 is a known region of the ascending trigemino-cortical pathway, and local reorganization could account for some of the altered sensory experiences reported between migraine attacks. Based on this work, we envisage the ASL methodology could have direct clinical research applications in migraine, with the potential for assessing perfusion status before or during migraine attacks, and in response to therapy [59].
